# Mechanical Effects on K_ATP_ Channel Gating in Rat Ventricular Myocytes

**DOI:** 10.1371/journal.pone.0063337

**Published:** 2013-05-14

**Authors:** Haixia Huang, Lifang Liang, Ping Liu, Hua Wei, Frederick Sachs, Weizhen Niu, Wei Wang

**Affiliations:** 1 Department of Physiology and Pathophysiology, School of Basic Medical Sciences, Capital Medical University, Beijing, China; 2 Department of Physiology and Biophysics, SUNY, Buffalo, New York, United States of America; University of Tampere, Finland

## Abstract

Cardiac K_ATP_ channels link metabolism with electrical activity. They are implicated in arrhythmias, secretion of atrial natriuretic peptide and protection of the heart from hypertrophy and failure. These processes may involve mechanosensitivity. K_ATP_ channels can be activated by mechanical stimulation and disrupting the cortical actin increases the activity. We propose that K_ATP_ channels are modulated by local bilayer tension and this tension is affected by cortical F-actin. Here we measured K_ATP_ background activity and stretch sensitivity with inside-out patches of rat ventricular myocytes before and after disrupting F-actin. Disrupting F-actin potentiated background activity but did not influence the slope sensitivity in the semilog relationship of *NP_o_* vs. suction that is a measure of the change in dimensions between closed and open states. Thus actin alters prestress on the channel probably by parallel elastic sharing of mean cortical tension with the bilayer.

## Introduction

Adenosinetriphosphate (ATP)-sensitive K^+^ (K_ATP_) channels are expressed at high density (10/patch or >1/µm^2^) in rat cardiac myocytes [1]. They sense intracellular ATP or the ATP/ADP ratio and link metabolism to electrical activity [2], [3]. The channels are activated at low ATP as caused by metabolic stresses such as ischemia or hypoxia. Activation of a small fraction of K_ATP_ channels can significantly hyperpolarize the cell and reduce action potential duration [1], [4] and Ca^2+^ influx, and sequentially protect myocardium from hypoxia.

Van Wagoner first reported that K_ATP_ channels were mechanosensitive [5] and activated by hypotonic stimulation in atrial myocytes [6]. Saegusa et al. demonstrated that the atrium and atrial cells from Kir6.2 knockout mice secreted more atrial natriuretic peptide (ANP) in response to stretch than those of the wild-type [7]. Shi et al. showed that K_ATP_ channels were involved in the regulatory volume decrease in rat ventricular myocytes [8]. In addition, K_ATP_ channels are postulated to protect the heart from hypertrophy and failure induced by pressure-overload [9].

There are two working models for the gating mechanism of mechanosensitive channels (MSCs). One is the tethered model where stress is transferred directly to the channel through intracellular and extracellular fibrous proteins as in the tip links of hair cells of the cochlea [10]. The second is the bilayer model where stress is transmitted as tension through the bilayer. This mechanism is known to apply to mechanosensitive channels from Escherichia coli [11], [12], [13], [14]. Stretch sensitivity of eukaryotic channels to bilayer tension has not yet been demonstrated although a recent report suggests that hPIEZO1 channels feel the same force as the bacterial channels [15]. A study on stretch-activated K^+^ channels shows that the channel could be pressure-dependently activated in patches from both the control atrial myocyte and the hypotonically induced membrane bleb with minimal cytoskeleton [16], indicating that cortical F-actin is not a prerequisite for channel activation mechanically but shares cortical tension and helps protect the channel from excess activation.

K_ATP_ channels are known to be modulated by the cytoskeleton. Kline [17] reported that ankyrin B, a cytoskeletal adapter protein, was required for the Kir6.2 trafficking process and it modulated K_ATP_ channels through interaction with a Kir6.2 C-terminal motif. A single residue mutation in this motif (E322K) decreased the targeting of Kir6.2 to the cell membrane (loss-of-function) and its ATP sensitivity (gain-of-function). The same results were confirmed in the cardiac myocytes from ankyrin-B+/− mice where both ankyrin B and the Kir6.2 expression and I_KATP_ were lower, although the channel open probability was higher than in wild type mice [18]. Losing the interaction with the ankyrin B appears to enhance stretch-channel opening. This result is consistent with previous studies showing that disruption of actin with cytochalasin and DNase?increased activation of K_ATP_ in cardiac myocytes [19]. In the present study, we tested whether cortical F-actin might play a role as a parallel elastic component to the lipid membrane. We disturbed F-actin with various drugs and measured the background activity in patches (the activity without applied stretch) and the response to applied stretch. Disruption of F-actin potentiated the background activity but did not influence the slope of the semilog relationship between *NP_o_* and suction pressure.

## Materials and Methods

### Preparation of Single Ventricular Myocytes

All animal experimental procedures were approved by the Institutional Animal Care and Use Committee of the Capital Medical University, Beijing, China, and performed in accordance with “Regulations for the Administration of Affairs Concerning Experimental Animals (the State Science and Technology Commission, China, 1988)”.

Ventricular myocytes were enzymatically isolated by retrograde perfusion of the heart via Langendorff apparatus. Briefly, Sprague-Dawley rats (female, 250∼300 g) were injected with heparin (2500 unit/kg) and then sodium pentobarbital (50 mg/kg). When the rat was anaesthetized, the heart was quickly excised and rinsed with ice-cold Tyrode’s solution containing (in mM): NaCl 133.5; KCl 4.0; MgSO_4_ 1.2; NaH_2_PO_4_ 1.2; CaCl_2_ 1.8; Glucose, 11.0; HEPES 10.0; Taurine, 30.0; and titrated to pH 7.4 with NaOH. The heart was perfused sequentially with Tyrode’s solution for 5 min to remove blood, with Ca^2+^-free Tyrode’s solution for 5∼6 min, and then with Ca^2+^-free Tyrode’s solution containing 1 mg/ml collagenase (type II, Worthington, Freehold, USA) and 1 mg/ml bovine serum albumin (BSA, fraction V, Sigma-Aldrich, St. Louis, USA) for 30∼40 min. All perfusion solutions were equilibrated with 95% oxygen and 5% CO_2_ and maintained at 37°C. After collagenase treatment the ventricles were cut into small pieces and mechanically dissociated into single cells. The harvested cells were stored at 4°C in a solution, containing (in mM): KCl 40.0; KOH 80.0; KH_2_PO_4_ 25.0; MgSO_4_ 3.0; Glucose 10.0; Taurine 20.0; Glutamic Acid 50.0; EGTA 1.0; HEPES 10.0; buffered at pH 7.4 with KOH.

### Single Channel Recording and Analysis

K_ATP_ currents were recorded in inside-out mode at room temperature. The standard pipette and bath solutions contained (in mM): L-aspartic acid potassium 140.0; HEPES 10.0; MgCl_2_ 2.0; EGTA 5.0; buffered at pH 7.2 with KOH. For determining the reversal potential of the channel, the concentration of potassium aspartate in the pipette solution was reduced to 70 and 35 mM with replacement 70 and 105 mM NaCl to maintain isosmotic conditions. Pipettes made from borosilicate glass (BF150-110-10, Sutter Instrument Co., Novato, USA) had resistances of 6.0∼8.0 MΩ. The current was amplified with an Axopatch 200B amplifier (Molecular Devices, Foster City, CA, USA) and digitized at 20 kHz using a Digidata 1322A and pClamp 9.2 software (Molecular Devices, Foster City, CA, USA). Suction was applied to the pipette in parallel with a water-filled manometer with a resolution of 5 mm H_2_O. The pressure could be manually reset in 1 sec.

Data were analyzed with QUB (www.qub.buffalo.edu) and pClamp 9.2 at 2 kHz. The level of K_ATP_ channel activity was quantitated by the average number of open channels *(NP_o_*) defined as *ΣnP_n_*, where *N* represents the number of K_ATP_ channels in the patch, *P_o_* the probability of one channel, *n* (*1≤ n ≤ N*) the level of the channel opening, *P_n_* the probability when *n* identical channels are open simultaneously [20]. Inward currents were routinely displayed downward.

### Drugs

Glibenclamide, cytochalasin B (cytoB) and phalloidin (Sigma-Aldrich, St. Louis, USA) were made as stock solutions in dimethyl sulphoxide (DMSO, Sigma-Aldrich, St. Louis, USA) and kept at −20°C until required, then diluted in bath solution to a final concentration of 10 µM, 20 µM and 10 µM, respectively. The final DMSO concentration did not exceed 0.1% and control studies showed that DMSO at that concentration had no measurable effect on K_ATP_ activity. Stock solutions of MgATP, thymosin β4 and G-actin (Sigma-Aldrich, St. Louis, USA) in deionized water were stored at −20°C and diluted for use with bath solution to concentrations of 0.2 mM, 50 µg/ml and 100 µg/ml. All drugs were applied to the intracellular side.

### Statistical Analysis

Data are expressed as mean ± SE. Student’s paired *t-*test compared data obtained before and after an intervention. A two-way analysis of variance (ANOVA) followed by a Tukey's Multiple Comparisons Test compared data from different pressures. For the patch-clamp data, n indicates the number of patches. The statistical significance was set at *P*<0.05.

## Results

### Identification of K_ATP_ Channels in Ventricular Myocytes

**Figure 1:**
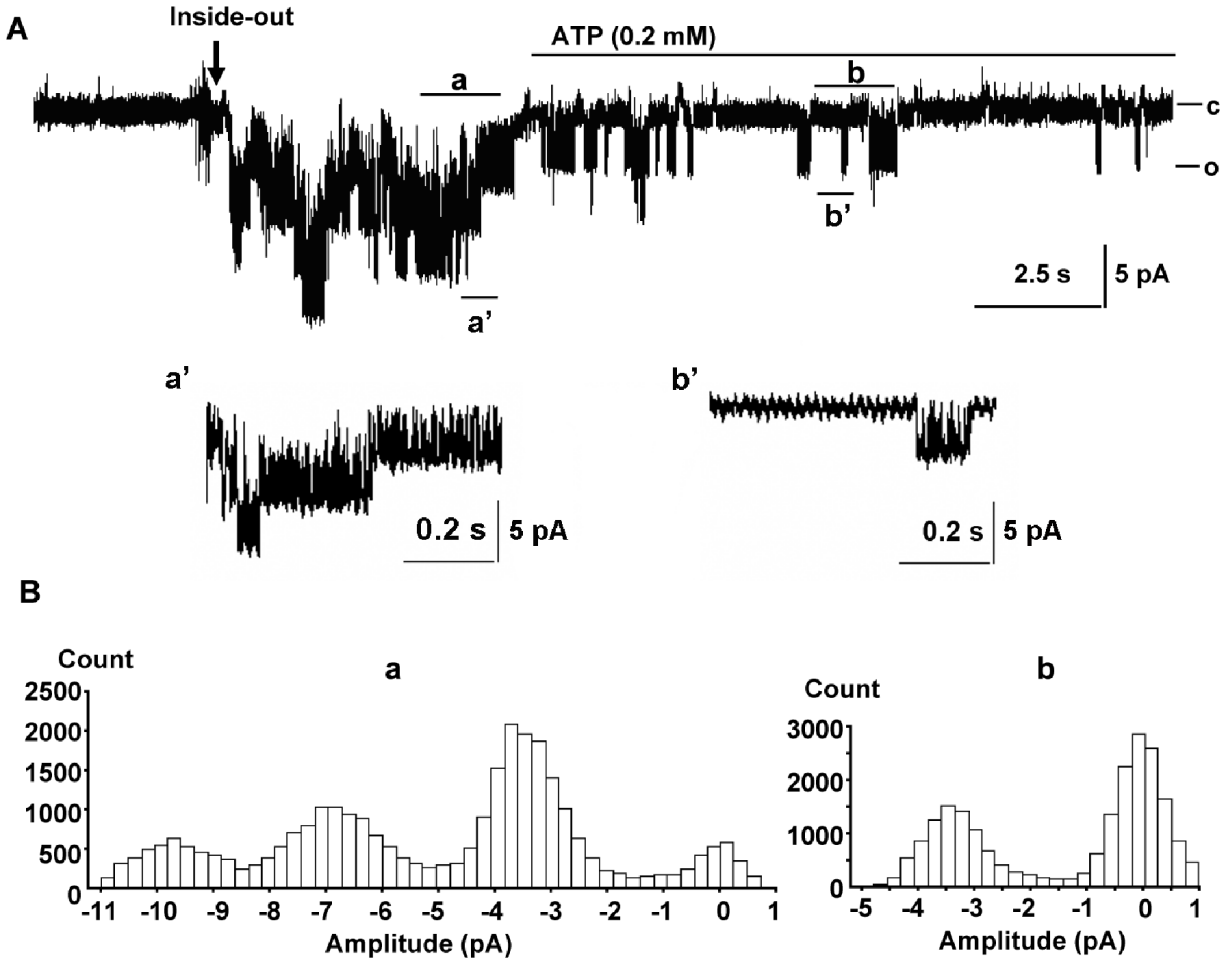
K_ATP_ currents in an inside-out patch from a rat ventricular myocyte. A: Currents were continuously recorded at −60 mV in symmetrical solutions. Channel activity increased upon excision and decreased with MgATP (0.2 mM). Inward current is downward. The lower current traces (a’ and b’) show the channel activities at a higher time resolution picked from the record at times marked with the corresponding letters in the upper traces. B: All-points histograms (a and b) of the data segments tagged with a and b in panel A, showing the same unitary current before and during MgATP.

The currents were first confirmed to be coming from K_ATP_ channels. Cell-attached patches had little channel activity because of the inhibition by cytoplasmic ATP. Upon excision, channel openings appeared (Figure 1A). MgATP (0.2 mM) applied to the internal side of the patch reduced channel activity. *NP_o_* was 0.372±0.046 after excision and decreased to 0.065±0.030 after the addition of MgATP (n = 10, *P*<0.001). All-points histograms before and during MgATP showed the same unitary currents (Figure 1Ba and Bb). The reversal potential of single channel currents was checked with pipette solutions containing 140, 70 and 35 mM K^+^ and a bath solution containing 140 mM K^+^. The corresponding currents reversed at 0, −17 and −31 mV, close to estimates of E_K_ from the Nernst equation. The mean conductance was slightly inwardly rectifying with 42±0.8 pS at +60 mV and 55±1.5 pS at −60 mV (n = 7) in symmetrical K^+^. After addition of 10 µM glibenclamide to the bath, *NP_o_* was reduced from 0.181±0.060 to 0.009±0.004 (n = 7, *P*<0.05). Based on the conductance, sensitivity to ATP and glibenclamide, weak inward rectification and reversal potentials close to the K^+^ equilibration potential, the currents arose from K_ATP_ channels.

### The Mechanosensitivity of K_ATP_ Channels in Ventricular Myocytes

**Figure 2:**
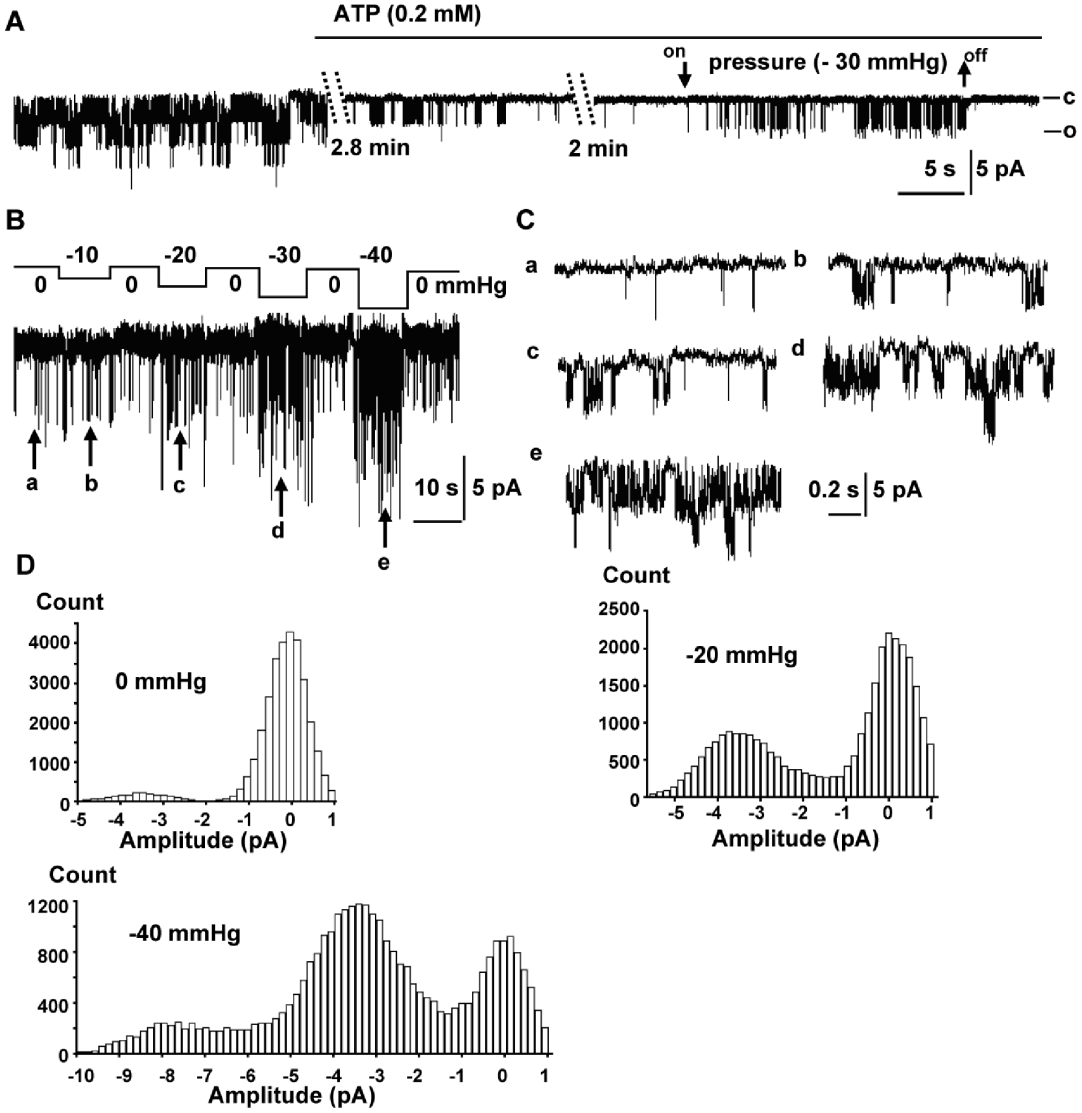
K_ATP_ response to suction. Current was recorded at −60 mV in symmetrical solutions at the indicated pressures. A: Two data segments of 2.8 min with 2 min of intervening data omitted to show steady state behavior. K_ATP_ was inhibited by 0.2 mM ATP and activated by suction. B: K_ATP_ response to pressure ladders. C: Traces a to e show longer term activity and are marked with corresponding letters in B. D: All-points histograms were constructed using the data segments (1.6 sec each) in B at 0, −20 and −40 mm Hg, respectively.

Figure 2A shows K_ATP_ activity following excision from another myocyte. To test the specificity of the current and reduce rundown we routinely added MgATP (0.2 mM) to the bath solution. As shown in Figure 1A, addition of MgATP decreased the open probability. K_ATP_ activity increased significantly with 30 mmHg suction (Figure 2A) and the activity ceased immediately after the relief of suction showing reversibility.

The pressure sensitivity of the currents from −10 to −40 mm Hg was determined with four 10-second suction pulses separated by 10-second relaxation periods. The maximum number of current levels and the open probability reversibly increased as a function of the applied pressure (Figure 2B and C). *NPo* was 0.107±0.042, 0.182±0.042, 0.248±0.064, 0.371±0.064 and 0.560±0.094 at 0, −10, −20, −30 and −40 mmHg, respectively (n = 7). *NPo* at −30 and −40 mm Hg was higher than that before suction significantly (*P*<0.05). The data are similar to those from atrial cells [5]. All-points histograms at 0, −20 and −40 mm Hg (Figure 2D) show the same single channel current (*P*>0.05, n = 7; data not shown) so that unitary conductance was not modified by stretch.

### Effects of the State of the Actin Cytoskeleton on Mechanosensitivity

#### Effects of CytoB

To analyze the data quantitatively we used an exponential to approximate the foot of the Boltzmann relationship [21]. The relationship between *NP_o_* and suction pressure was fitted with *NP_o_* = *Ae^kx^*, where *x* is the pressure, *A* is the background activity (the intercept) and *k* is the slope (in units of 1/mm Hg) in the semilog relationship of *NP_o_* vs. pressure.

**Figure 3:**
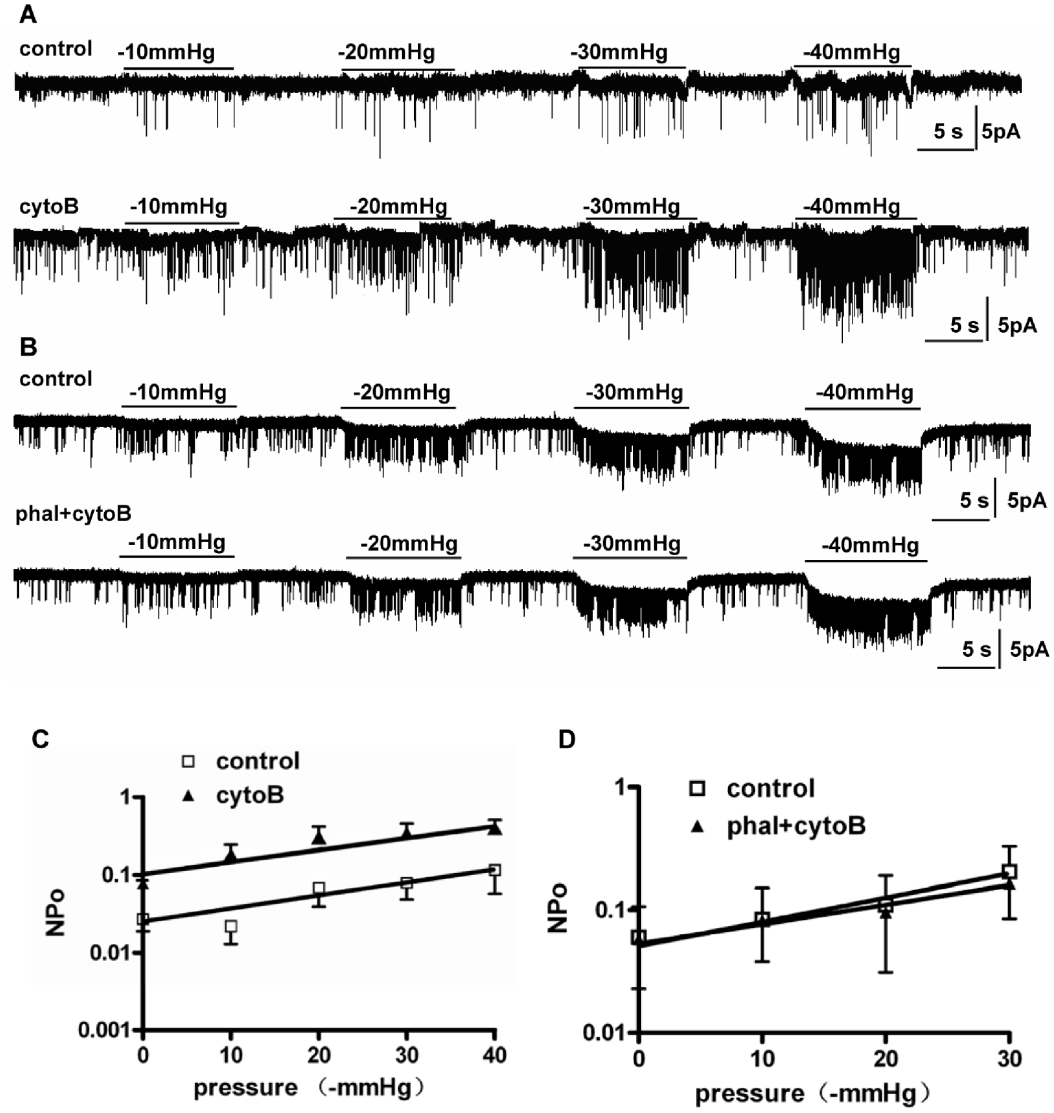
Effect of cytoB on the response to pressure steps. A: Recordings from a patch before and after cytoB treatment. B: Recordings in another patch before and after exposure to phalloidin (10 µM) followed by cytoB (20 µmol/L) (phal+cytoB). C: The semilog plots of *NP_o_*-pressure relationship before and after treatment with cytoB. The two semilog plots are parallel while the intercept is increased by cytoB (n = 6; *P*<0.05) suggesting cytoB increases the background activity only. D: The semilog plots of *NP_o_*-pressure relationship before and after treatment with phalloidin followed by cytoB (phal+cytoB). The two semilog graphs coincides and the increment in background activity induced by cytoB was abolished by application of phalloidin in advance (n = 5; *P*>0.05).

Figure 3A shows records from a patch in response to incremental pressure stimuli before and after 5-min treatment with cytoB (20 µM). The background activity increased after treatment with cytoB. The channels showed pressure-dependent responses in both conditions. The statistical data are plotted on a semilog scale in Figure 3C that shows two parallel *Log*(*NP_o_*)-pressure lines fit with 0.022*e* ^0.042*x*^ (*R* ^2^ = 0.85; n = 6) for the control and 0.105*e* ^0.040*x*^ (*R* ^2^ = 0.86; n = 6) for cytoB treatment, respectively. The mean background *NP_o_* (the intercept) increased from 0.027±0.008 to 0.078±0.012 after cytoB treatment (n = 6; *P*<0.05). Channels could be activated in a pressure-dependent fashion both before and during cytoB treatment. In addition, the slopes (the *k* value) for the two lines were almost equal (0.042 vs. 0.040), indicating that disrupting cortical F-actin did not influence *k*, the slope sensitivity.

As a control for the effect of cytoB we preapplied the actin-stabilizing reagent,phalloidin, to oppose the subsequent addition of cytoB. Figure 3B shows that pretreatment with phalloidin (10 µM, 20 min before cytoB) inhibited the effect of cytoB (20 µM, 5 min) on background activity, but not on slope *k*. Figure 3D shows two fitting functions, *NP_o_ = *0.057*e* ^0.040*x*^ (*R* ^2^ = 0.96; n = 5) for the control and *NP_o_ = *0.056*e* ^0.033*x*^ (*R* ^2^ = 0.95; n = 5) for the treatment with phalloidin followed by cytoB. The background activity was 0.060±0.047 for the control and 0.058±0.035 for the treatment with phalloidin plus cytoB (n = 5; *P*>0.05) corresponding to slopes of 0.040 vs. 0.033. Thus, cortical actin influences the K_ATP_ background activity but not the slope sensitivity *k* that is an *intrinsic* mechanical property of the channel and related to dimensional changes between shut and open.

#### Effects of Thymosin-β4

**Figure 4:**
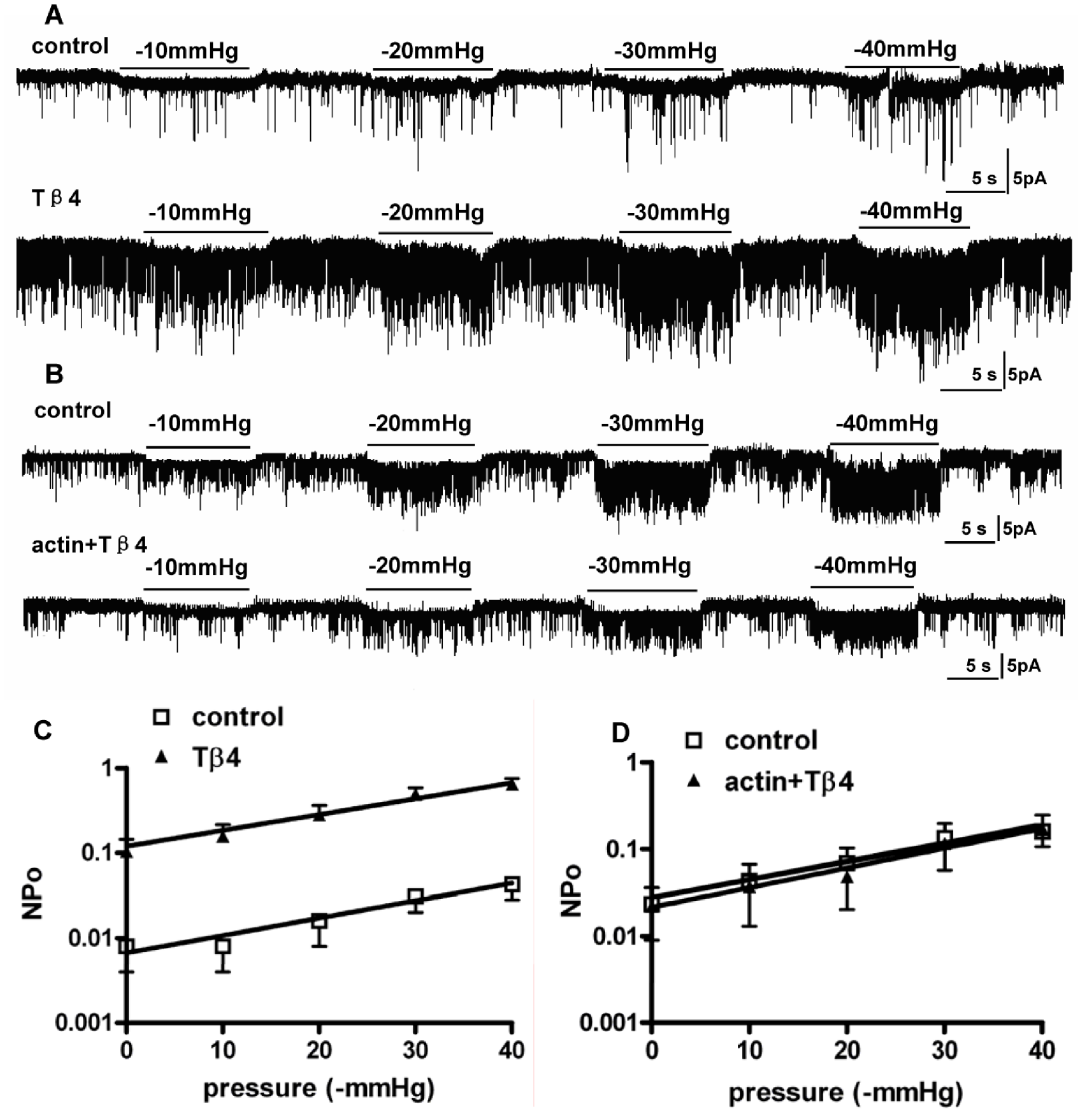
Effect of thymosin-β4. A: Recordings from the same patch before (control) and after thymosin-β4 (Tβ4, 50 µg/ml) treatment. B: Recordings from another patch before (control) and then treatment with G-actin (100 µg/ml) followed by thymosin-β4 (50 µg/ml) (actin+Tβ4). C: The semilog plot of *NP_o_*-pressure relationships before (control) and after treatment with thymosin-β4 (Tβ4). The two fits are parallel while the *NP_o_* is increased by thymosin-β4 (n = 7; *P*<0.05). D: The semilog plotting of *NP_o_*-pressure relationships before (control) and after treatment with G-actin followed by thymosin-β4 (actin+Tβ4). The two semilog plots are parallel and the intercept isn’t increased by thymosin-β4 due to combined application of G-actin (n = 5; *P*>0.05).

The polymerization of G-actin to form F-actin is a dynamic equilibrium. Thymosin-β4 is a G-actin sequestering protein and promotes F-actin degradation by shifting the equilibrium towards the monomeric form. Figure 4A shows a record from a patch under incremental mechanical challenges before and after a 10-min treatment with thymosin-β4 (50 µg/ml), exhibiting a remarkable enhancement of the background activity. However, the response to graded mechanical stimuli was reserved. Statistical data were fitted with *NP_o_ = *0.007*e* ^0.047*x*^ (*R* ^2^ = 0.94; n = 7) for the control and *NP_o_ = *0.104*e* ^0.048*x*^ (*R* ^2^ = 0.99; n = 7) for the thymosin-β4 treatment, respectively. Similar to the effects of cytoB (Figure 3A), depolymerizing F-actin increased the background activity from 0.008±0.004 to 0.104±0.040 (n = 7, *P*<0.05) but did not change the slope *k* (0.047 vs 0.048).

To test for specificity of thymosin-β4 effects we applied G-actin (100 µg/ml) together with thymosin-β4 (50 µg/ml) in the bath solution. As shown in Figure 4B, this eliminated the effect of thymosin-β4 on background activity (0.023±0.013 vs 0.022±0.014; n = 5; *P*>0.05). The data were fit with *NP_o_ = *0.025*e* ^0.050*x*^ (R^2^ = 0.98; n = 5) for the control and *NP_o_ = *0.021*e* ^0.052*x*^ (R^2^ = 0.97; n = 5). The fitting gave approximate intercepts as well as slopes, demonstrating that depolymerizing F-actin raises the background activity but not the slope *k* (Figure 4C and 4D). According to Figure 3 and 4, *NP_o_* can be clearly divided into three components: the basal activity (may be ligand-dependent for ADP, ATP or PIP_2_), the actin-modulated activity and the suction-dependent activity.

## Discussion

### Mechanosensitivity of K_ATP_ Channel in Ventricular Myocytes

K_ATP_ channels are dominantly composed of Kir6.2 with SUR1 in the atrium and Kir6.2 with SUR2A in the ventricle [22]. In rat atrial cells K_ATP_ channels are mechanosensitive [5], [6] and now we have shown that is also true in the ventricle. In this study the highest *NP_o_* was 0.644±0.084 (n = 7) at −40 mmHg suction after treatment with thymosin-β4. If we assume a density of 10 channels per patch in rat ventricular myocytes for pipettes with a resistance of 2∼8 MΩ (assuming ∼1 channel/µm^2^) [1], the highest *Po* in our study would be about one order of magnitude lower (∼0.064), a small dynamic range unless the saturation level was seriously underestimated. An *apparent* mechanosensitivity may be defined as the steepness of the *NP_o_*-pressure curve, *d(NP_o_)/dx* = *Ake^kx^* or equivalently *d(NP_o_)/dx = kNP_o._* where parameter *A* represents the background activity (including the basal activity plus an increment after actin disruption), *k* is the intrinsic sensitivity and *x* is the pressure, or in ideal case, the local tension. If local bilayer tension increases with actin disruption, the steepness of the curve should increase. The mechanical microenvironment for K_ATP_ in a patch is not known but must include the bilayer, with possibly multiple phases, and the cortical skeleton. The cytoskeleton shares stress with the bilayer so we would anticipate that disrupting actin would increase stress in the bilayer. The steepness of the *NP_o_*-pressure curve (*Ake^kx^*) and the slope *k* reflect two distinct meanings: one is the *apparent* sensitivity that is dependent on both external conditions (ligands, actin status and pressure) and the *intrinsic* property (slope *k*) of the channel and the other is the *intrinsic* sensitivity depending on the energy difference between closed and open states.

In this study we have also examined the *NP_o_*-pressure curve for the rat atrial K_ATP._ The data fitted with *NP_o_* = 0.115*e* ^0.040*x*^ (*R* ^2^ = 0.99; n = 7; not shown in the result part). The slope sensitivity closely matches that of ventricular myocytes implying that the K_ATP_ dimensional changes between closed and open are similar. Values of *k* in the literature range from 0.06∼0.08 for stretch-activated potassium channels (TREK-1-like channels) in rat atrial myocytes [16] and 0.03 (*R* ^2^ = 0.93, n = 6) for Ca^2+^-activated large-conductance potassium channel (BK_Ca_, also sensitive to stretch) in colonic smooth muscle [23]. When the absolute sensitivity has been measured *k* should provide an atomic measure of the dimensional changes of the channel between closed and open [15].

### Bilayer Model of Mechanical Gating

The patches are always under tension due to adhesion of the membrane to the glass in the gigaseal [24]. Loss of a parallel elastic element (F-actin) will transfer that stress to the bilayer and the increased bilayer stress can modulate the channels, thereby increasing background activity. The sensitivity *d(NP_o_)/dx* is an *apparent* sensitivity that is affected by actin status so the data is consistent with local tension being actin-dependent.

During the cardiac cycle, the sarcolemma is subjected to a variety of forces including tension and shear forces along the longitudinal axes and compression of T-tubules. The costamere is a major part of the cortical cytoskeleton and is presumably involved in the distribution of forces. Costamere protein complexes can be divided into three main groups: the spectrin/ankyrin/transporter (and some channels, such as voltage-gated sodium channels and K_ATP_ channels), the dystrophin/dystroglycan and the vinculin/talin/α-actinin/integrin complexes. All three groups connect to the Z-disk through F-actin [25]. This linkage is thought to protect the sarcolemma from disengaging from the Z-disk during contraction. Disrupting the cytoskeleton will influence the distribution of cortical stress and that will change the bilayer tension that is probably responsible for activation of the channels.

Clusters of K_ATP_ channel are localized in the Z-grooves [26] and transverse tubules of the ventricular myocytes [18] and bind to ankyrin B that connects to the cortical spectrin-actin network [27]. According to a report by Naruse et al. [28] that mechanosensitivity of BK_Ca_ channel in chick embryonic heart was dependent on the interaction between a key amino acid in the channel C-terminal (Ala674) and submembranous components, it is possible for K_ATP_ that the tension might be transferred to the K_ATP_ C-terminal to gate the channel through ankyrin B-spectrin-actin network. However, disrupting ankyrin B-Kir6.2 binding by a C-terminal mutation (E322K) of Kir6.2 caused an K_ATP_ gain-of-function [17] and decreased ankyrin-B expression in ankyrin-B+/− mice enhanced open probability [18]. Both results do not support the ankyrin tether model for K_ATP_. They are consistent with previous reports that disrupting the cortical F-actin network with cytoB or DNase I potentiated K_ATP_ activity [19] and that may also suggest a transfer of more cortical tension to the bilayer. The present study supports that interpretation.

### Physiological and Pathophysiological Significance

In the present study, we have shown that K_ATP_ activity is determined by three factors, ligands (such as ATP concentration), cortical actin status and stretching. In an experimental model of *Commotio Cordis* with low-energy chest wall impact, blocking K_ATP_ with glibenclamide significantly decreased the incidence of ventricular fibrillation and the amplitude of the ST segment [29], consistent with a sharp activation of K_ATP_ and a shortening of action potential and refractory period. This study suggests that K_ATP_ contributes to fibrillation. Significantly, there is no K_ATP_ current in ventricular myocytes from the Kir6.2 knockout mouse and yet the knockout heart is more vulnerable to pressure overload [9]. There were contrary reports arguing that K_ATP_ channels play a negligible role modulating in vivo cardiac contraction or arrhythmia in normal and failing heart [30]. However, increasing evidence has shown that K_ATP_ plays an important role in feedback from metabolic signals (ischemic insult, hemodynamic overload, sympathetic surge) to the excitability control mechanism [3], [31]. Mechanosensitivity might provide an additional mechanism to protect the heart, especially during ischemia or hypoxia when the cytoskeleton might degrade [32] and the myocyte is swollen [33]. Two explanations involving K_ATP_ are common for explaining the higher susceptibility to cardiac hypertrophy and failure in Kir6.2 knockout mouse: loss of the mechanosensitivity and energetic decoding [9]. K_ATP_ activation by any means leads to hyperpolarization and a reduction in Ca^2+^ influx by affecting Ca^2+^ channel gating. K_ATP_ channels are required for proper functioning in cardioprotection under ischemic insult and in the response to acute and chronic (pathophysiologic) hemodynamic loads [34]. These effects are likely to be the result of modulation by both metabolic signals and mechanical stress.
